# Case Report: Pseudo-prune belly syndrome after early fetal bladder decompression: implications for the pathogenesis of prune belly spectrum disorders

**DOI:** 10.3389/fruro.2026.1919700

**Published:** 2026-08-25

**Authors:** Alberto Parente, Mercedes Fariñas, Monica Miño, Francisca Sonia Molina

**Affiliations:** 1 Pediatric Surgery Department, Reina Sofia University Hospital, Córdoba, Spain; 2 Pediatric Department, Reina Sofia University Hospital, Córdoba, Spain; 3 Gynecology and Obstetrics Department, Reina Sofia University Hospital, Córdoba, Spain; 4 Department of Obstetrics and Gynecology, Hospital Universitario Clínico San Cecilio, Granada, Spain

**Keywords:** hydronephrosis, prenatal treatment, prune belly anomaly, prune belly pseudo-syndrome, vesicoamniotic shunting

## Abstract

**Background:**

Prune Belly Syndrome (PBS) is a rare congenital disorder characterized by abdominal wall deficiency, urinary tract dilatation, and bilateral cryptorchidism. Its pathogenesis remains controversial, with both developmental and mechanical theories proposed. Whether early fetal decompression influences the phenotypic expression of PBS has not been established.

**Case presentation:**

We report a male fetus diagnosed at 12 weeks’ gestation with severe megacystis and bilateral hydroureteronephrosis. A Somatex^®^ vesicoamniotic shunt was placed at 16 weeks because of progressive lower urinary tract obstruction. Prenatal follow-up demonstrated complete resolution of upper urinary tract dilatation and persistent bladder decompression throughout pregnancy. After birth, the patient presented bilateral cryptorchidism and mild abdominal wall weakness but preserved renal morphology and function. Voiding cystourethrography showed high-grade bilateral vesicoureteral reflux, and diagnostic cystoscopy excluded posterior urethral valves and urethral atresia, supporting the diagnosis of Pseudo–Prune Belly Syndrome (PPBS). At 6 months of follow-up, the patient remains asymptomatic, with no urinary tract infections, spontaneous bilateral testicular descent, stable abdominal wall weakness, normal renal growth, and preserved renal function.

**Conclusion:**

This case illustrates a favorable early outcome following prenatal vesicoamniotic shunting in a fetus with severe lower urinary tract obstruction who subsequently developed an attenuated phenotype consistent with PPBS. Although causality cannot be inferred from a single observation, these findings are consistent with the fetal outlet obstruction hypothesis and raise the possibility that early fetal decompression may influence phenotypic expression in selected patients. Further studies are required to clarify the role of prenatal intervention in the pathogenesis and clinical spectrum of PBS.

## Introduction

Prune belly syndrome (PBS), also known as Eagle–Barrett syndrome, is a rare congenital disorder classically characterized by the triad of abdominal wall musculature deficiency, urinary tract dilatation, and bilateral cryptorchidism (1, 2). Its estimated incidence ranges from 1 in 30,000 to 50,000 live births, with more than 95% of affected patients being male (1–3). Incomplete phenotypes, commonly referred to as pseudo-prune belly syndrome (PPBS), account for approximately 3%–5% of cases and exhibit variable combinations of the classical features (4, 5).

The pathogenesis of PBS remains incompletely understood. Two main hypotheses have been proposed: a primary mesodermal developmental defect affecting the abdominal wall, urinary tract, and genital structures, and the fetal outlet obstruction hypothesis, in which prolonged bladder distension causes secondary abdominal wall deficiency, impaired testicular descent, and progressive urinary tract dilatation (1, 2, 6). More recently, pathogenic variants involving genes such as CHRM3, FLNA, and HNF1B have suggested a developmental contribution in some patients. Overall, current evidence supports a multifactorial origin involving developmental, obstructive, and genetic factors (2).

Prenatal ultrasonography has enabled earlier diagnosis of PBS spectrum disorders through the detection of megacystis, hydroureteronephrosis, oligohydramnios, and renal dysplasia (1, 2, 6). Because renal impairment and pulmonary hypoplasia are the main determinants of prognosis, fetal intervention has been proposed in carefully selected cases with severe lower urinary tract obstruction (2, 4, 7).

Vesicoamniotic shunting (VAS) is the most widely used fetal decompression technique, aiming to preserve renal development, maintain amniotic fluid volume, and reduce pulmonary morbidity (7). Although encouraging renal and survival outcomes have been reported, its effect on the phenotypic expression of PBS remains uncertain. Herein, we present a fetus with severe lower urinary tract obstruction treated with early VAS who subsequently developed an attenuated phenotype consistent with PPBS, raising the possibility that prenatal decompression may influence disease expression without establishing a causal relationship.

## Case report

**Figure 1:**
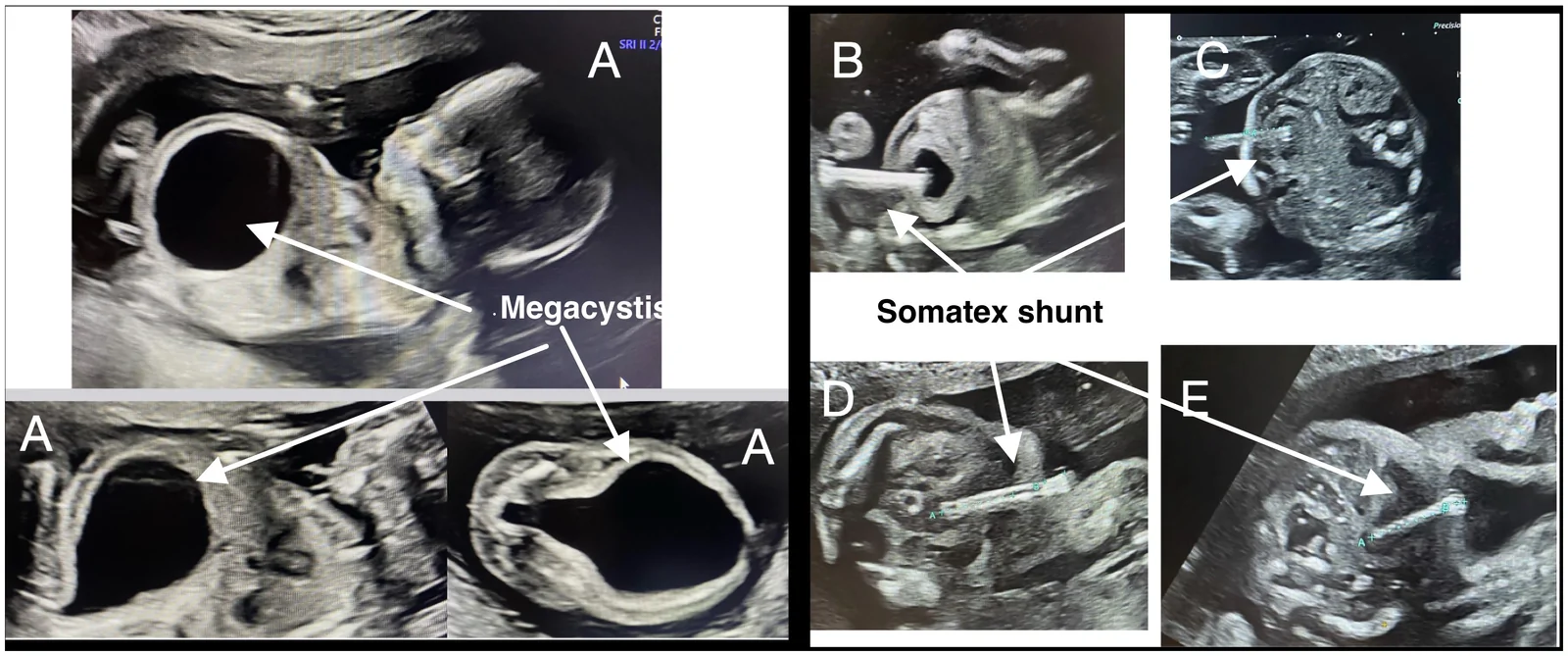
Fetal ultrasound. **(A)** Week 15 of gestation, megacystis (40 × 43 mm). **(B)** Week 16 with vesicoamniotic shunt. **(C)** Week 22. **(D)** Week 27. **(E)** Week 32.

A pregnant woman was referred to our fetal medicine unit at 12 weeks’ gestation after routine prenatal ultrasonography revealed severe bilateral hydroureteronephrosis associated with marked fetal megacystis (20 × 22 mm). At the time of diagnosis, the amniotic fluid volume was within normal limits. At 15 weeks of gestation, worsening of the megacystis (40 × 43 mm) with a keyhole was observed (**Figure 1**). The case was discussed at a multidisciplinary prenatal meeting, where fetal bladder decompression was considered because of the significant bladder enlargement and the potential risk of compressive complications.

The patient was referred to a tertiary fetal therapy center, where a vesicoamniotic shunt was placed at 16 weeks’ gestation using a Somatex^®^ intrauterine shunt. Subsequent weekly ultrasound examinations demonstrated complete resolution of the bilateral hydroureteronephrosis, persistent decompression of the bladder, and appropriate shunt positioning throughout pregnancy (**Figure 1**). Gestation progressed uneventfully, except for the worsening of the mother’s diabetes mellitus.

Delivery was induced at 36 weeks because of complications related to diabetes. The newborn did not exhibit respiratory distress and required no ventilatory support. Urethral catheterization was attempted shortly after birth; however, passage of the catheter proved difficult and was ultimately achieved under ultrasound guidance using a hydrophilic guidewire. After confirmation of appropriate bladder drainage, the vesicoamniotic shunt was removed without complications.

**Figure 2:**
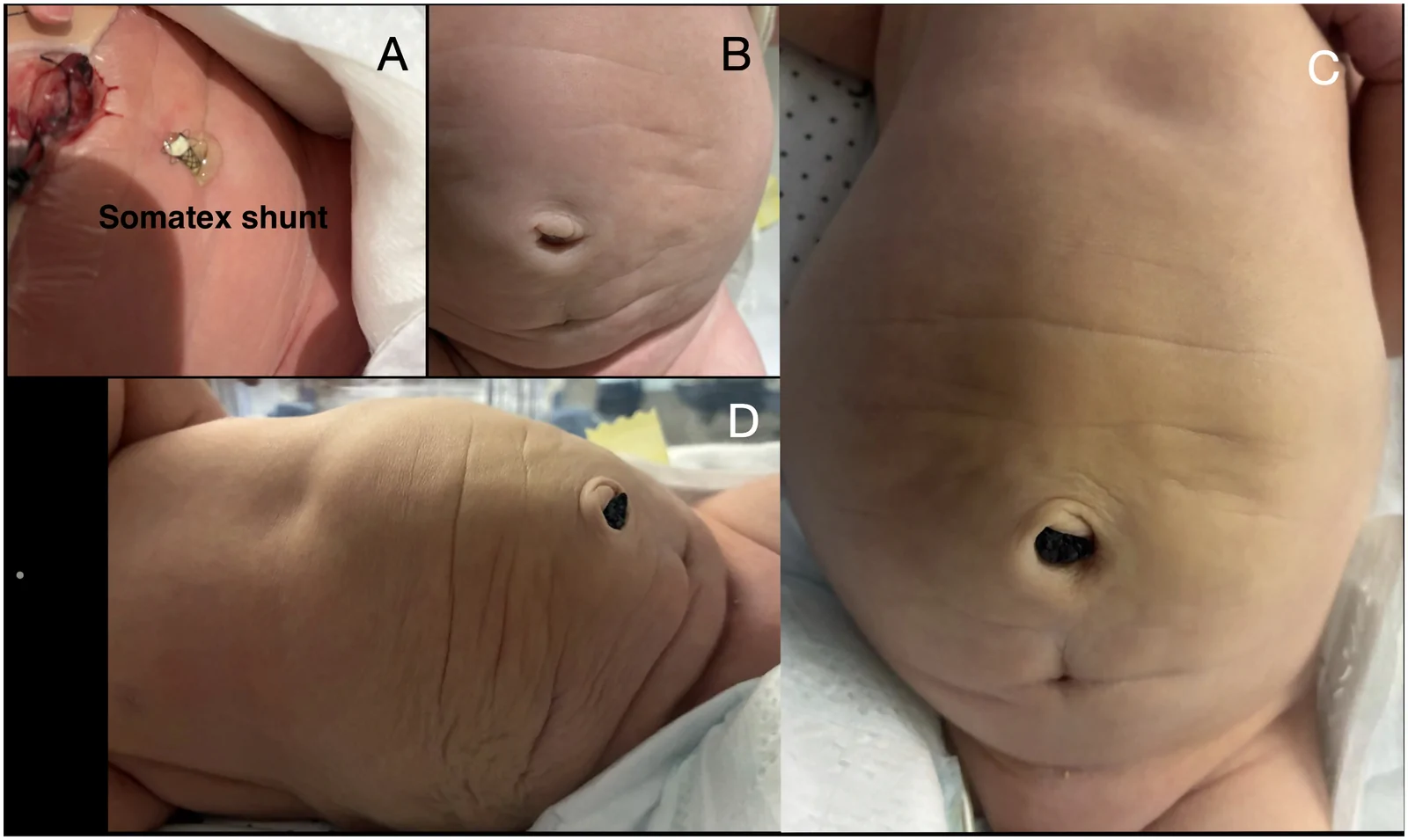
Abdominal examination. **(A)** At birth, with the drain still in place. **(B)** Third day of life. **(C)** Seventh day of life. **(D)** One month of life.

Physical examination revealed bilateral cryptorchidism, with a non-palpable right testis and a left testis palpable within the inguinal canal. Mild abdominal wall laxity was noted at birth and became progressively more evident during the neonatal period (**Figure 2**).

**Figure 3:**
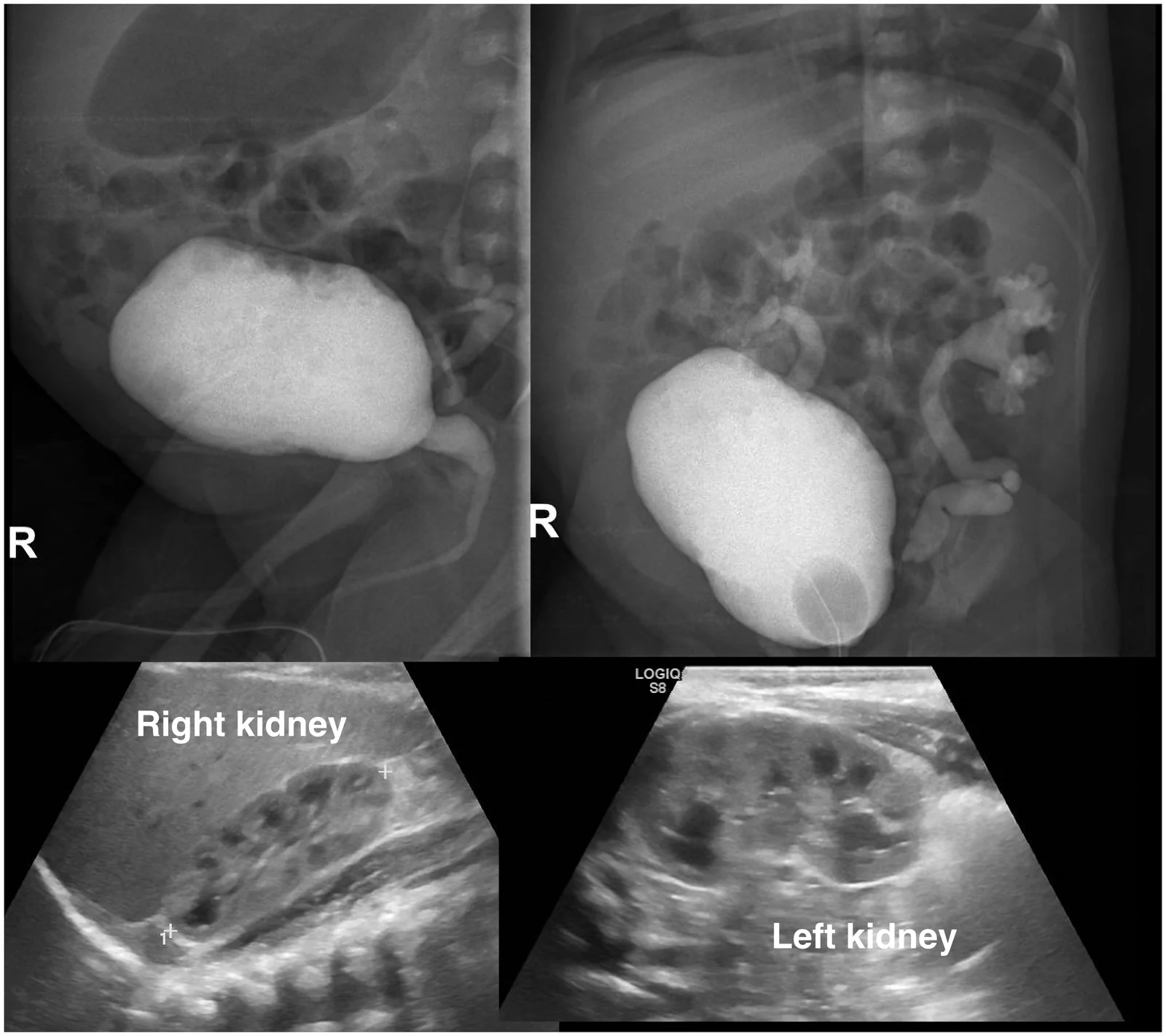
Voiding cystography with high-grade vesicoureteral reflux. Ultrasound on the 7th day of life.

Renal and urinary tract ultrasonography performed at 48 h and again at 7 days of life demonstrated complete resolution of the prenatal hydroureteronephrosis, with normal renal morphology and no evidence of upper urinary tract dilatation (**Figure 3**). Serum renal function parameters were within normal limits. A dimercaptosuccinic acid (DMSA) renal scan showed preserved bilateral renal cortical function without scarring.

Voiding cystourethrography revealed a markedly enlarged bladder associated with bilateral high-grade vesicoureteral reflux (grades IV–V) and mild urethral dilatation (**Figure 3**). Based on these findings, diagnostic cystoscopy was performed. Endoscopic evaluation excluded posterior urethral valves and demonstrated mild narrowing of the prostatic urethra, a large-capacity bladder, and orthotopic ureteral orifices with a refluxing appearance.

The urethral catheter was removed at 15 days of life, after which spontaneous voiding was normal. At 3 months of age, spontaneous descent of the right testis into the inguinal canal and of the left testis into the scrotum was observed. Minimally progressive abdominal wall weakness remained clinically evident. The patient continues on continuous low-dose antibiotic prophylaxis and has experienced no urinary tract infections during follow-up.

At the 6-month follow-up, the patient remains asymptomatic, with no episodes of urinary tract infection. Bilateral testicular descent has been completed spontaneously, and the abdominal wall weakness has remained stable without progression. Renal ultrasound demonstrates appropriate bilateral renal growth without hydronephrosis, and renal function remains within normal limits (**Table 1**).

**Table 1:** Timeline.

Gestational age	Event
12 weeks	Megacystis + bilateral hydroureteronephrosis
15 weeks	Progressive megacystis (40 × 43 mm)
16 weeks	Somatex vesicoamniotic shunt
22 weeks	Resolution of hydroureteronephrosis
27 weeks	Normal fetal follow-up
32 weeks	Persistent bladder decompression
36 weeks	Delivery
Birth	PPBS phenotype
3 months	Partial spontaneous testicular descent
6 months	Complete testicular descent, normal renal function

## Discussion

PPBS is a rare and incompletely characterized condition within the spectrum of PBS. While classical PBS is defined by the triad of abdominal wall deficiency, bilateral cryptorchidism, and urinary tract dilatation, PPBS encompasses patients with partial or atypical manifestations of these features (1, 4, 5). Owing to its low incidence and phenotypic heterogeneity, the natural history and underlying pathophysiological mechanisms of PPBS remain poorly understood (2, 5).

The distinction between incomplete PBS and PPBS remains controversial, and terminology has not been standardized. In our patient, the diagnosis of PPBS was considered more appropriate because, despite the presence of abdominal wall hypoplasia and bilateral cryptorchidism, severe urinary tract abnormalities resolved almost completely following early prenatal decompression. Consequently, the classic triad of PBS was not fully expressed after birth. We acknowledge that some authors may classify similar presentations as incomplete PBS; therefore, the terminology should be interpreted within the context of the heterogeneous spectrum of the disease (2, 5).

The present case is remarkable because severe fetal megacystis and bilateral hydroureteronephrosis were detected during the second trimester and successfully treated by VAS, resulting in complete prenatal resolution of upper urinary tract dilatation and preservation of renal function after birth. Although VAS has been primarily advocated to prevent progressive renal damage and pulmonary hypoplasia in selected fetuses with lower urinary tract obstruction, its long-term impact on the phenotypic expression of PBS spectrum disorders remains unknown (7).

The differential diagnosis of fetal megacystis includes posterior urethral valves, urethral atresia, megacystis–microcolon–intestinal hypoperistalsis syndrome, cloacal malformations, and other syndromic or genetic conditions. In our patient, postnatal cystoscopy excluded posterior urethral valves and urethral atresia, while the absence of associated gastrointestinal or cloacal abnormalities made other syndromic conditions unlikely. Taken together with the prenatal imaging findings and the postnatal phenotype, these findings favored the diagnosis of an attenuated phenotype consistent with PPBS rather than another congenital cause of fetal lower urinary tract obstruction.

The etiology of PBS and PPBS continues to be debated. Two major theories have traditionally been proposed. The mesodermal developmental defect hypothesis suggests a primary embryological abnormality affecting the abdominal wall musculature, urinary tract, and genital structures (1, 2). In contrast, the fetal outlet obstruction hypothesis attributes the syndrome to prolonged bladder distension during fetal life, leading to secondary abdominal wall stretching, urinary tract dilatation, and impaired testicular descent (1, 2, 7). Neither theory independently explains the broad clinical variability observed among affected patients, and current evidence suggests that PBS likely results from a multifactorial interaction between developmental, obstructive, and genetic factors (2).

Recent advances in molecular genetics have provided further insight into the etiology of PBS and its incomplete variants. Mutations involving genes associated with smooth muscle differentiation and genitourinary development, including CHRM3, FLNA, and HNF1B, have been identified in a subset of patients (2). However, most patients remain genetically unexplained, suggesting substantial genetic heterogeneity and the contribution of additional developmental and environmental factors (2). Phenotypic expression within the broader PBS spectrum rather than a distinct pathological entity. Genetic testing was not performed in this patient. Although several genetic abnormalities have recently been associated with PBS, the absence of molecular evaluation limits our ability to exclude an underlying genetic contribution to the phenotype. This limitation should be considered when interpreting the proposed pathogenetic implications of this case.

An important aspect of the present case is its potential contribution to the ongoing debate regarding the pathogenesis of PBS spectrum disorders. At 12 weeks’ gestation, the fetus presented with severe megacystis and massive bilateral hydroureteronephrosis, indicating marked and prolonged urinary tract distension during a critical period of fetal development. Following early VAS, bladder decompression was successfully achieved, resulting in complete resolution of upper urinary tract dilatation and preservation of normal renal development. The clinical evolution observed in our patient is consistent with the fetal outlet obstruction hypothesis and raises the possibility that prolonged fetal bladder distension could contribute substantially to the phenotypic expression of PBS spectrum disorders. However, conclusions regarding causality cannot be drawn from a single clinical observation.

Interestingly, despite the initial severity of the urinary tract abnormalities, the patient did not develop the complete classical PBS phenotype (8, 9). Instead, postnatal findings were more consistent with an incomplete or pseudo-Prune Belly presentation, characterized by mild abdominal wall laxity, cryptorchidism, preserved renal function, and absence of the severe urinary tract deterioration typically observed in classical PBS. Our findings are consistent with the fetal outlet obstruction hypothesis and raise the possibility that early prenatal decompression may modify the subsequent phenotypic expression of the disease. However, causal relationships cannot be established from a single clinical observation.

According to mechanical theory (10–12), prolonged fetal bladder distension leads to progressive stretching and thinning of the developing abdominal wall musculature. The resulting reduction in intra-abdominal pressure may subsequently impair normal testicular descent, contributing to the development of cryptorchidism (1, 2, 6). The present case could be consistent with this model. Despite severe fetal megacystis during early gestation, prompt decompression through VAS resulted in the preservation of renal morphology and function, spontaneous partial testicular descent during infancy, and the development of only a mild PPBS phenotype rather than complete PBS. These findings could suggest that bladder overdistension may play an active pathogenic role in the development of abdominal wall deficiency and cryptorchidism rather than representing a secondary consequence of an underlying developmental abnormality.

Conversely, if PBS were exclusively caused by a primary mesodermal developmental defect affecting the abdominal wall, urinary tract, and gonadal structures from the outset, one would expect prenatal urinary diversion to have little or no influence on the final phenotype. Under such circumstances, decompression of the urinary tract would be unlikely to alter the developmental trajectory of the syndrome. Although a single case cannot refute the mesodermal hypothesis, the favorable evolution observed in our patient raises the possibility that mechanical factors contribute substantially to disease severity and that timely fetal decompression may attenuate the progression toward the complete PBS phenotype.

Although follow-up is still limited, the favorable clinical evolution observed during the first 6 months of life—including the absence of urinary tract infections, normal renal growth and function, spontaneous completion of testicular descent, and stable abdominal wall weakness—is consistent with a favorable early outcome following prenatal decompression. Nevertheless, longer-term follow-up is required to assess bladder function, continence, renal function, and overall functional outcomes.

## Conclusion

This case suggests that early VAS may be associated with attenuation of the phenotypic severity of PBS in selected fetuses with severe lower urinary tract obstruction. Although a single case cannot definitively establish causality, this case is consistent with the fetal outlet obstruction hypothesis and raises the possibility that early fetal decompression may influence the phenotypic expression of PBS spectrum disorders. Larger prospective studies are required before definitive conclusions regarding causality or treatment effect can be drawn.

## Data Availability

The datasets presented in this article are available upon request to the author. Requests to access the datasets should be directed to Alberto Parente.
